# Analytical characterization and clinical evaluation of an enzyme-linked immunosorbent assay for measurement of afamin in human plasma^☆^

**DOI:** 10.1016/j.cca.2013.08.016

**Published:** 2013-10-21

**Authors:** Benjamin Dieplinger, Margot Egger, Christian Gabriel, Werner Poelz, Elisabeth Morandell, Beata Seeber, Florian Kronenberg, Meinhard Haltmayer, Thomas Mueller, Hans Dieplinger

**Affiliations:** a Department of Laboratory Medicine, Konventhospital Barmherzige Brueder Linz, Linz, Austria; b Red Cross Transfusion Service of Upper Austria, Linz, Austria; c Institute for Applied System Sciences and Statistics, University of Linz, Linz, Austria; d Department of Gynecological Endocrinology and Reproductive Medicine, Innsbruck Medical University, Innsbruck, Austria; e Division of Genetic Epidemiology, Department of Medical Genetics, Molecular and Clinical Pharmacology, Innsbruck Medical University, Innsbruck, Austria; f Vitateq Biotechnology GmbH, Innsbruck, Austria

**Keywords:** ELISA, enzyme-linked immunosorbent assay, CRP, C-reactive protein, PCT, procalcitonin, IL-6, interleukin-6, BNP, B-type natriuretic peptide, eGFR, estimated glomerular filtration rate, PBS, phosphate-buffered-saline, CLSI, Clinical and Laboratory Standards Institute, RT, room temperature, CV, coefficient of variation, RCV, reference change value, HF, heart failure, COPD, chronic obstructive pulmonary disease, Tumor marker, Diagnosis, C-reactive protein, Interleukin-6, Prognosis, Vitamin E

## Abstract

**Background:**

Comparative proteomics has recently identified afamin, the newest member of the albumin gene family, as a potential biomarker for ovarian cancer. The aim of this study was the analytical and clinical evaluation of a sandwich enzyme-linked immunosorbent assay for the determination of afamin in human plasma.

**Methods:**

We evaluated precision, linearity, and detection limit of the assay, analyte stability and biological variability, determined reference values and quantified afamin concentrations in various diseases.

**Results:**

Within-run and total coefficients of variation were < 10%. The method was linear across the tested measurement range. Detection limit was 7 mg/L for the assay. The analyte was stable for 24 h at room temperature, for 48 h at 4 °C, and for at least one year at − 20 °C and − 80 °C. The reference change value for healthy individuals was 24%. Age- and sex-independent reference values in healthy blood donors were 45–99 mg/L (median 68 mg/L). In the clinical assay evaluation afamin plasma concentrations were modestly decreased in patients with heart failure. Patients with pneumonia or sepsis exhibited markedly decreased afamin plasma concentrations. However, patients with chronic renal disease or chronic obstructive pulmonary disease showed no difference in afamin plasma concentrations as compared to healthy individuals. Correlation analyses revealed an inverse association between afamin and inflammatory biomarkers.

**Conclusions:**

The afamin assay meets quality specifications for laboratory medicine. The results of the clinical assay evaluation revealed novel insights with respect to afamin as a potential negative acute phase protein and should encourage further studies.

## Highlights

• The aim of this study was the analytical and clinical evaluation of a human afamin ELISA.
• The afamin assay meets the needs of quality specifications of laboratory medicine.
• Afamin exerts a good in vitro stability which is important of preanalytical issues.
• The RCV was 24% and reference values in healthy blood donors were 45–99 mg/L.
• We revealed novel insights with respect to afamin as a potential negative acute phase protein.

## 1 Introduction

The glycoprotein afamin was discovered in 1994 by two independent research groups in humans [1] and in rats [2] as the fourth member of the albumin gene family which is located on chromosome 4q11–q13 in humans [1]. Araki et al. [3] demonstrated that afamin is identical to α-1T-glycoprotein, a protein first reported as a tryptophan-poor α-1T-protein almost 50 years ago [4]. In contrast to albumin, afamin is highly glycosylated and exerts a molecular mass of 75 kDa [5]. Afamin has four or five potential N-glycosylation sites, and treatment with N-glycanase reduces the apparent molecular mass of afamin to 65 kDa [1]. Like other members of the albumin multigene family, afamin consists of three structural domains indicating 17 Cys–Cys disulfide bridges [1].

Afamin is predominantly expressed in the liver and secreted into the bloodstream; minor expressions have been described also in human brain [6], heart, kidney, testis and ovary (www.proteinatlas.org). Afamin has been reported to bind vitamin E, especially α-tocopherol and γ-tocopherol, two of the most important forms of vitamin E, in vitro and in vivo and to possess multiple binding sites for both tocopherol isomers [5,7].

Comparative proteomics has previously identified afamin as a potential biomarker for ovarian cancer [8]. These findings were confirmed with immunoblotting and a quantitative immunoassay for afamin. Patients with ovarian cancer displayed significantly decreased plasma concentrations of afamin by comparison to healthy controls. These results were later validated in an independent larger study of patients with ovarian cancer [9] and, very recently, extended by showing significant associations between afamin plasma concentrations and clinical outcomes (response to therapy and survival rates) [10]. In contrast, afamin concentrations were not found to be decreased in benign gynecological conditions including endometriosis [9,11].

We have developed a fully automated immunoassay for the quantitative measurement of afamin in human plasma. The aim of this study was to evaluate the analytical and clinical performance of this sandwich enzyme-linked immunosorbent assay (ELISA) for afamin.

## 2 Material and methods

### 2.1 Study protocol

Most portions of this work were derived from a single-center study performed at the St. John of God Hospital in Linz, Austria. Remaining portions were performed at Innsbruck Medical University, Innsbruck, Austria. The study protocol was approved by the ethics committees of St. John of God Hospital in Linz and Innsbruck Medical University, in accordance with the Declaration of Helsinki. All study participants gave informed consent. Blood was obtained by conventional venipuncture avoiding venous stasis. Using VACUETTE® polyethylene terephthalate glycol blood collection tubes (Greiner Bio-One, Kremsmünster, Austria), one anticoagulated EDTA blood sample and one whole-blood sample were collected and plasma or serum obtained by low-speed centrifugation for 10 min. Each study participant underwent measurement of afamin, C-reactive protein (CRP), procalcitonin (PCT), interleukin-6 (IL-6), creatinine, albumin, and B-type natriuretic peptide (BNP). Further specifications of the study protocol have been reported previously [12] and are outlined in the respective paragraphs of this comprehensive Methods section.

### 2.2 Afamin measurement

Afamin was measured with photometric enzyme-linked immunosorbent assay (ELISA) on a fully automated BEP® 2000 instrument (Siemens Healthcare Diagnostics, Vienna, Austria). The afamin ELISA is a custom-made double-antibody sandwich assay (MicroCoat GmbH, Bernried, Germany) using an affinity-purified polyclonal rabbit anti-human-afamin antibody for coating 96-well microtiter plates and the enzyme-conjugated monoclonal mouse anti-human afamin antibody N13 for detection [5,7]. For a detailed description on the primary afamin protein and antibodies used for the afamin assay, see Supplementary data. Antibody N13 was conjugated with horse-radish peroxidase according to standard protocols and purified by size-exclusion chromatography prior to use. The biotinylated coating antibody was bound to streptavidin-coated plates, followed by blocking unspecific binding by incubation with 0.1% casein in phosphate-buffered-saline (PBS). After removing blocking solution microtiter plates were sealed and kept at 4 °C until use.

All subsequent incubation steps were carried out at room temperature (18–25 °C). Antibody-coated microtiter plates were washed three times with 300 μL of washing buffer [Tween 20 (1/2000, vol/vol) in PBS, pH 7.3] and incubated with 100 μL blank, standards, and plasma samples after a standard 1:1000 dilution with assay buffer (0.1% casein in PBS, pH 7.3), washing buffer under constant agitation at 750 rpm for 60 min.

A secondary plasma standard with an afamin concentration of 80 mg/L, calibrated with purified human afamin, was used in a serial dilution for the present study. Seven standards with the following final dilutions were used for each assay: S1 = 0.200 mg/L, S2 = 0.100 mg/L, S3 = 0.050 mg/L, S4 = 0.025 mg/L, S5 = 0.0125 mg/L, S6 = 0.0063 mg/L, S7 = 0.0031 mg/L.

Microtiter plates were then washed three times with 300 μL washing buffer and incubated with 100 μL horse-radish-peroxidase-conjugated monoclonal antibody N13 (diluted 1:2000 in assay buffer to a final concentration of 1 μg/mL) under agitation at 700 rpm for 60 min. After washing another three times with 300 μL washing buffer, 100 μL of the chromogenic substrate solution 3,3′,5,5′-tetramethylbenzidine (TMB, Blue Star, Adaltis, Rome, Italy) was added and the plates were finally incubated under agitation at 700 rpm for 15–30 min. After sufficient color development, the reaction was stopped by adding 50 μL 1 N H_2_SO_4_, followed by brief agitation and photometrical analysis at a wavelength of 450 nm within 5 min after adding stop solution. Afamin concentrations were calculated with a linear regression model (x axis and y axis log transformed) after subtraction of the average blank.

In the present work, we used EDTA-plasma samples for all afamin determinations, except for the study comparing plasma and serum samples. Afamin was measured in each sample in duplicates on the same microtiter plate and the mean was calculated from these two determinations.

### 2.3 Further biochemical analyses

Serum concentrations of CRP, PCT, IL-6, creatinine, albumin, and BNP plasma concentrations were measured as reported previously by our group [12]. Estimated glomerular filtration rate was calculated using the Chronic Kidney Disease Epidemiology Collaboration (CKD-EPI) equation: 141 × min (creatinine [mg/dL]/κ,1)^α^ × max (creatinine [mg/dL]/κ,1)^− 1.209^ x 0.993^age^ × 1.018 [if female] × 1.159 [if black]; where κ = 0.7 if female and κ = 0.9 if male, α = − 0.329 if female and α = − 0.411 if male [13].

### 2.4 Precision study

To evaluate the precision of the afamin assay in our laboratory, we performed a replication study according to the Clinical and Laboratory Standards Institute (CLSI; formerly NCCLS) Guideline EP5-A [14]. Three pooled patient plasma samples were aliquoted into twenty 1.5-mL plastic tubes for each concentration level and frozen at − 80 °C. We analyzed these samples in duplicate in one run per day for 20 days on a single BEP® 2000 instrument within two months of blood collection. Within-run and total analytical imprecision (CV_A_) was calculated with the CLSI single-run precision evaluation test [14].

### 2.5 Evaluation of linearity

We also evaluated the linearity of the afamin assay according to the CLSI Guideline EP6-A [15] using six different analyte concentrations. Fresh plasma samples were used to prepare a high- and a low-concentration pool spanning the measurement range of the assay. We then performed a direct dilution series with the low- and high-concentration patient sample pools in the following volume ratios (low-concentration pool + high-concentration pool): pool 1, low only; pool 2, 0.8 low + 0.2 high; pool 3, 0.6 low + 0.4 high; pool 4, 0.4 low + 0.6 high; pool 5, 0.2 low + 0.8 high; and pool 6, high only. Each concentration was measured in triplicate and the default criteria were set at 5% for repeatability and 5% for nonlinearity.

### 2.6 Detection limit

The detection limit for the afamin assay was determined by assaying a diluted sample from a healthy individual in replicates of 20 and was calculated as three standard deviations added to the resulting mean value of the diluted sample. The diluted sample was prepared by mixing a fresh plasma sample in a 1:15 ratio with sample dilution buffer. This prediluted plasma sample was then treated in the same way as all other patient samples. The 20 replicates were assayed on the same microtiter plate.

### 2.7 Stability study

We evaluated the stability of afamin concentrations (measured with ELISA) after storing plasma samples from 15 patients with various diseases at room temperature (RT, 18–25 °C), 4 °C, − 20 °C, and − 80 °C. The stability of afamin plasma concentrations at RT and 4 °C was evaluated after 4, 12, 24, 48 and 72 h, after seven days and after one month. The stability of afamin at − 20 °C and at − 80 °C was evaluated after 24 h, after seven days, after one and six months, and after one year. Baseline afamin plasma concentrations were determined immediately after blood collection. At the same time, plasma samples were aliquoted into 1.5-mL plastic tubes and stored in appropriate amounts at the specified temperatures. Subsequently, these aliquots were used to measure afamin concentrations after the specified time intervals. Mean recovery (i.e., residual immunoreactivity) of afamin expressed in absolute values (i.e., absolute recovery) and in percent of the initial value (i.e., relative recovery) for the given time interval of storage was calculated. The default criterion for analyte stability was set at 95%; this means afamin was considered stable at a distinct storage condition as long as the median relative recovery was ≥ 95%.

### 2.8 Study of biological variation

Twenty-two apparently healthy members of our laboratory staff (12 males and 10 females; age range, 22–59 years) were recruited for determination of components of biological variation (intra- and inter-individual CV) for afamin as described previously [12] and as outlined in the Supplementary data. Once every week for six weeks, plasma was collected from each volunteer under standardized conditions to minimize sources of pre-analytical variation. Intra-individual biological CV (CV_I_), inter-individual biological CV (CV_G_), and the reference change value (RCV) were calculated according to the methods described by Fraser and Harris [16]. The within-run CV_A_ value was retrieved from our precision study.

### 2.9 Reference values for afamin

Reference values for the afamin assay were derived from 528 healthy blood donors at the Red Cross Organization in Linz, Austria, as reported previously [12] and as outlined in the Supplementary data.

### 2.10 Afamin concentrations in serum and plasma

In order to compare afamin concentrations between serum and plasma, one serum sample and one EDTA-plasma sample were obtained from each of eight apparently healthy members of our laboratory staff (four males, four females) after an overnight fast.

### 2.11 Afamin concentrations in fasting and non-fasting individuals

In 25 patients (19 males and 6 females) with various diseases (eight of them with type 2 diabetes mellitus) a plasma sample was drawn at 07:00 a.m. following an overnight fast. Thereafter, all patients had a standardized breakfast (730 kcal for patients without diabetes, and 522 kcal for those with diabetes). The next blood draw for afamin determination was at 11:00 a.m., after which all patients had a standardized lunch (800 kcal for patients without diabetes, and 716 kcal for those with diabetes). The final blood draw was at 02:00 p.m. after lunch.

### 2.12 Diurnal profile of afamin in healthy females

The circadian variation of afamin concentrations was investigated by analyzing afamin concentrations in 14 healthy non-pregnant females recruited at the Department of Gynecological Endocrinology and Reproductive Medicine, Innsbruck Medical University. Blood was collected in the non-fasting state, starting at 08:00 a.m. and proceeding until 08:00 a.m. the next morning (i.e., 08:00 a.m., 12:00 p.m., 04:00 p.m., 08:00 p.m., 12:00 a.m., 04:00 a.m., and 08:00 a.m.).

### 2.13 Afamin during menstrual cycle

In order to evaluate variations in afamin plasma concentrations during the menstrual cycle, afamin was analyzed in 18 non-pregnant females recruited at the Department of Gynecological Endocrinology and Reproductive Medicine, Innsbruck Medical University. Plasma samples were obtained in the early follicular phase (cycle day 3 to 5), around the time of ovulation (cycle day 10 to 12, pre-ovulatory), and in the mid-luteal phase (cycle day 21 to 23).

### 2.14 Clinical evaluation study

To examine afamin plasma concentrations in various diseases we recruited 15 inpatients each with ‘heart failure (HF) without co-morbidity’, ‘pneumonia without co-morbidity’, ‘chronic obstructive pulmonary disease (COPD) without co-morbidity’, ‘HF with co-morbidity of pneumonia’, ‘renal disease without co-morbidity’, or ‘sepsis’ as reported previously [12] and outlined in the Supplementary data. We used this approach to examine conditions suspected of influencing afamin concentrations and to disentangle possible confounders. As a control group we included the healthy individuals used for the biological variation study with the results obtained from their first blood withdrawal.

### 2.15 Correlation of afamin with other biomarkers

To further elucidate the correlation between afamin and CRP, PCT, IL-6, albumin, and BNP we used plasma samples from 100 patients with a variety of diseases, as reported previously [12]. In order to evaluate the association between afamin and other biomarkers across a wide range of each in diseased patients, we recruited this convenient sample of patients with a main diagnosis of COPD (n = 25), heart failure (n = 22), renal disease (n = 16), pneumonia (n = 15), inflammatory bowel disease (n = 12), or pulmonary embolism (n = 10) leading to hospital admission.

### 2.16 Statistical analyses

Data were statistically analyzed with the SPSS 13.0 software (SPSS Inc.) and the MedCalc 12.7.0.0 package (MedCalc Software). Relationships between continuous variables were tested with the Spearman coefficient of rank correlation (r_s_). Comparisons of continuous variables between patient groups were performed with the Wilcoxon test, the non-parametric Mann–Whitney *U* test, the Friedman test, or the Kruskal–Wallis test, as appropriate. Obtained p values were not adjusted for multiple comparisons and are therefore descriptive only.

## 3 Results

The afamin assay had a within-run CV_A_ of 8.7% and a total CV_A_ of 9.9% at a mean concentration of 26 mg/L (pool 1, low), a within-run CV_A_ of 3.3% and a total CV_A_ of 6.2% at a mean concentration of 73 mg/L (pool 2, medium), and a within-run CV_A_ of 4.3% and a total CV_A_ of 5.4% at a mean concentration of 87 mg/L (pool 3, high).

The two high- and low-concentration plasma pools used to evaluate the assay linearity had afamin concentrations of 77 mg/L and 18 mg/L, respectively. The standard errors of regression (S_y,x_) and *t* tests of regression analyses showed that the first-order model fitted better than did the second- and third-order models: first-order model b_1_, S_y,x_ = 1.4, *t*-test = 62.7 (p < 0.001); second-order model b_2_, S_y,x_ = 1.3, *t*-test = 1.7 (p = 0.112); third-order model b_3_, S_y,x_ = 1.3, *t*-test = − 0.6 (p = 0.571). In addition, all default criteria were met. The method was thus linear within the measured range. The detection limit was 7 mg/L for the afamin assay.

Results of the stability study are shown in Table 1 with afamin values displayed as absolute concentrations and as percent recovery. When applying the pre-specified default criterion for analyte stability (i.e., 95% as detailed in Material and methods), afamin was stable for 24 h at RT, for 48 h at 4 °C, and for at least one year at − 20 °C and at − 80 °C.

As a result of the biological variation study, the overall median for afamin was 64 mg/L (range, 46–101 mg/L), the CVI was 7.9%, the CV_G_ was 14% and the RCV was 23.7%.

A histogram of afamin plasma concentrations in the healthy blood donors indicated a non-Gaussian distribution. Of the 528 healthy blood donors, 338 (64%) were male and 190 (36%) were female. Median afamin values did not differ significantly between male and female individuals (68 mg/L, range 33–113 mg/L vs. 68 mg/L, range 41–106 mg/L; p = 0.739). The median age of the healthy blood donors was 39 years (range 18–65 years). When stratifying the blood donors by age groups no significant differences in afamin plasma concentrations were observed across these groups (Kruskal–Wallis test, p = 0.104). Respective median afamin plasma concentrations according to age groups were: 18–24 years (n = 94), 71 mg/L (range 33–106 mg/L); 25–34 years (n = 131), 66 mg/L (range 40–98 mg/L); 35–44 years (n = 128), 66 mg/L (range 43–109 mg/L); 45–54 years (n = 127), 70 mg/L (range 33–113 mg/L); and 55–64 years (n = 48), 68 mg/L (range 38–102 mg/L). Further, Spearman coefficient of rank correlation analysis in the healthy blood donors revealed no association between afamin plasma concentrations and age (r_s_ 0.043, p = 0.346). Median eGFR of the healthy blood donors was > 90 mL/min/1.73 m^2^ (range 59–> 90 mL/min/1.73 m^2^). Furthermore, stratification of the blood donors by renal function did not reveal significantly different afamin plasma concentrations in individuals with an eGFR > 90 mL/min/1.73 m^2^ (n = 402) as compared to individuals with an eGFR < 90 mL/min/1.73 m^2^ (n = 126); median afamin plasma concentrations 68 mg/L (range 33–113 mg/L) vs. 68 mg/L, range (38–109 mg/L); Mann–Whitney *U* test, p = 0.827. We further calculated reference values by using a non-parametric percentile method (95%, double-sided). The reference interval for the afamin assay was 45–99 mg/L (median, 68 mg/L, range 33–113 mg/L; mean ± standard deviation, 69 mg/L ± 14).

No difference in median afamin concentrations was observed between serum and plasma samples from the same donors (73 mg/L, range 51–88 mg/L vs. 73 mg/L, range 50–89 mg/L; Wilcoxon test, p = 0.674).

When comparing afamin concentrations in 25 individuals in fasting and non-fasting state, median fasting afamin plasma concentration was 77 mg/L (range 45–104 mg/L) at 07:00 a.m., 76 mg/L (range, 47–112 mg/L; Wilcoxon test, p = 0.957 for comparison with fasting afamin) after breakfast at 11:00 a.m., and 78 mg/L (range 36–108 mg/L; Wilcoxon test, p = 0.819 for comparison with fasting afamin) after lunch at 02:00 p.m.

Evaluation of the diurnal profile of afamin in 14 healthy females by analyzing afamin plasma concentrations every 4 h from 08:00 a.m. to 08:00 a.m. the next morning revealed no significant differences among all seven time points (Friedman test, p = 0.516). In detail, median afamin plasma concentrations at different time-points were: 08:00 a.m., 71 mg/L (range 52–110 mg/L); 12:00 p.m., 71 mg/L (range 48–107 mg/L); 04:00 p.m., 73 mg/L (range 49–111 mg/L); 08:00 p.m., 68 mg/L (range 46–113 mg/L); 12:00 a.m., 70 mg/L (range 47–103 mg/L); 04:00 a.m., 66 mg/L (range 48–109 mg/L); and 08:00 a.m. the next morning 71 mg/L (range 51–111 mg/L).

Likewise, median afamin plasma concentrations did not differ significantly between the early follicular phase (65 mg/L, range, 40–110 mg/L), peri-ovulatory phase (62 mg/L, range 48–107 mg/L) and late luteal phase (67 mg/L, range 36–117 mg/L) in healthy, non-pregnant female probands (Friedman test, p = 0.946).

**Fig. 1:**
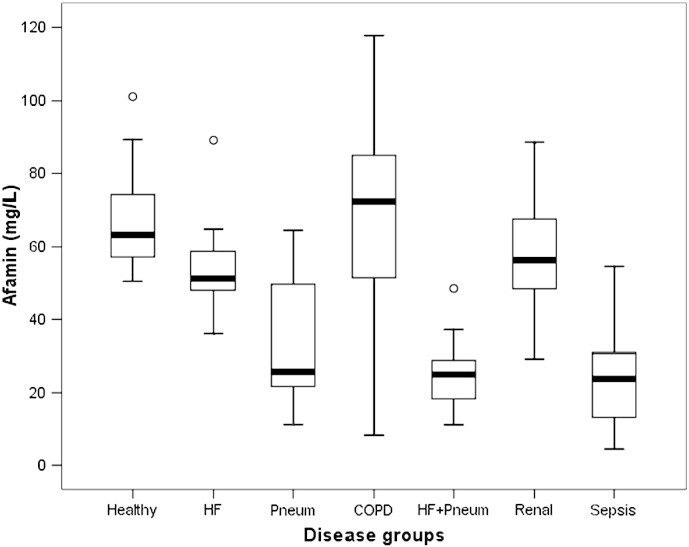
Distribution of afamin plasma concentrations in healthy individuals vs. diseased patients. Distribution of afamin plasma concentrations in healthy individuals (Healthy, n = 22) compared to patients with heart failure (HF, n = 15), with pneumonia (Pneum, n = 15), with chronic obstructive pulmonary disease (COPD, n = 15), with HF and co-morbidity of pneumonia (HF + Pneum, n = 15), with renal disease (Renal, n = 15), and with sepsis (Sepsis, n = 15), respectively.

Condensed data from the clinical evaluation study are given in Table 2. The distribution of afamin concentrations in healthy individuals and diseased patients is displayed in Fig. 1. The results indicate that there is no significant difference in afamin plasma concentrations between the control group and patients with COPD or renal disease. In contrast, mildly decreased afamin concentrations were obtained in patients with HF, and a more pronounced decrease in afamin concentrations was found in patients with pneumonia, HF plus pneumonia, or sepsis.

Of the 100 patients with various diseases, 83 were male and 17 were female, median age was 69 years (range 21–85 years) and the median afamin plasma concentrations were 50 mg/L (range 8–118 mg/L). Spearman coefficient of rank correlation analyses revealed a significant inverse association between afamin and CRP (r_s_ − 0.463, p < 0.001), PCT (r_s_ − 0.234, p = 0.019), and IL-6 (r_s_ − 0.471, p < 0.001), a borderline inverse association with BNP (r_s_ − 0.187, p = 0.063) and a significant positive association with albumin (r_s_ + 0.269, p = 0.007).

## 4 Discussion

We aimed to conduct an analytical and clinical evaluation study of a sandwich ELISA assay for the determination of afamin in human plasma. Our results demonstrate that the afamin assay meets the quality specifications for laboratory medicine. Furthermore, the results of our clinical assay evaluation are novel regarding the analysis of afamin in various diseases and should encourage further studies.

Within-run and total imprecision CV_A_ of < 9% and < 10%, respectively, were adequate, especially for an ELISA method. Furthermore, the assay was linear across the tested measurement range. In addition, the analyte afamin showed good stability data at different storage temperatures. As a result of this study, afamin can be considered stable for 24 h at RT, for 48 h at 4 °C, and for at least one year at − 20 °C and at − 80 °C. Thus, the analyte is well suitable for routine settings in laboratories, and also provides unproblematic conditions for sample shipment and storage.

The reference value study in healthy blood donors revealed that afamin plasma concentrations do not differ between males and females and are not associated with age or renal function in healthy blood donors. Thus, the age- and sex-independent reference values in healthy blood donors were 45–99 mg/L (median 68 mg/L, range 33–113 mg/L).

The biological variation study retrieved a CV_I_ of 7.9%, a CV_G_ of 14%, and a RCV of 23.7% in healthy individuals. The magnitude of RCV is rather low as compared with other analytes used in this study. For example, RCVs for BNP or PCT have been reported to exceed 50% in healthy individuals [17,18].

No relevant difference in afamin concentrations was seen for serum vs. plasma samples or for fasting vs. non-fasting state, indicating that serum and plasma samples are both suitable for afamin measurement and that afamin values can be determined independently of a patient's fasting state.

Further evaluation of the diurnal profile of afamin and the potential influence of menstrual cycle phase on afamin in healthy females revealed neither a circadian variation nor a relevant influence of menstrual phase on afamin plasma concentrations. Together with the data retrieved from the biological variation study, these findings underline the low variation of afamin over time, demonstrating good usability of afamin for serial measurements.

In the clinical assay evaluation study median afamin concentrations were only modestly decreased in patients with heart failure. Patients with pneumonia, heart failure and co-morbidity of pneumonia, as well as sepsis exhibited markedly decreased afamin concentrations. In contrast, in patients with chronic renal disease or COPD we found no difference as compared to healthy individuals. In this context it is noteworthy that also in the correlation analysis afamin showed a rather strong inverse association with the inflammatory biomarkers CRP and IL-6.

These findings are novel, especially the strong negative association between afamin and inflammatory disorders and inflammatory biomarkers. Even though pathophysiology so far is unclear, afamin might be a negative acute-phase protein.

Interestingly in this context, in our previous studies on the potential role of afamin as a biomarker for ovarian cancer we found only moderately decreased afamin plasma concentrations [8–10], e.g. median afamin plasma concentrations were 41 mg/dL in 215 patients with ovarian cancer [10] as compared to the rather low afamin concentrations we report for inflammatory disorders in the present study. Therefore, afamin plasma concentrations should be interpreted only in the context of comorbidities and inflammatory biomarker status.

Very little is known about the physiological or pathophysiological functions of afamin; large human epidemiological or animal model studies are still lacking.

In conclusion, the afamin assay meets the quality specifications for laboratory medicine. The analyte exerts good in vitro stability, which is important for preanalytical issues. The biological variation of afamin found in this study will serve as basis for further studies looking at serial measurements of afamin in different diseases. The results of the clinical evaluation study revealed novel insights with respect to afamin as a potential negative acute-phase protein, but raised several questions which can only be answered by future research.

## Grant/funding support

This study was partly supported by a grant from the Austrian Science Fund (P19969-B11) to H.D. Vitateq Biotechnology GmbH provided reagents for afamin measurement free of charge and did not play a role in (1) the design of the study; (2) data collection, analysis and interpretation.

## Disclosures

H. Dieplinger is owner and shareholder of Vitateq Biotechnology GmbH, a spin-off company of Innsbruck Medical University, holding several patents related to research described in this article. All other authors declare no conflicts of interest.

## Figures and Tables

**Table 1:** Summary of stability results for afamin: absolute and relative analyte recoveries at distinct time points for different storage conditions.

Analyte	Baseline	Storage	4 h	12 h	24 h	48 h	72 h	7 days	1 month	6 months	1 year
Afamin, mg/L	73 (52–98)	RT	71 (52–100)^‡^	73 (52–96)^‡^	72 (48 ± 98)^‡^	62 (48–90)***	64 (44–86)***	63 (47–85)***	66 (44–80)***	–	–
		4 °C	73 (49–100)^‡^	76 (52–98)^‡^	70 (50–95)^‡^	71 (54–94)^‡^	65 (43–91)*	66 (47–86)***	69 (47–82)*	–	–
		− 20 °C	–	–	72 (50–98)^‡^	–	–	72 (56–87)^‡^	71 (53–88)^‡^	70 (55–99)^‡^	71 (50–100)^‡^
		− 80 °C	–	–	73 (50–96)^‡^	–	–	73 (57–96)^‡^	75 (53–91)^‡^	72 (55–100)^‡^	75 (52–92)^‡^

Afamin, (%)	100	RT	101 (90–108)^‡^	97 (86–112)^‡^	98 (75–116)^‡^	89 (72–103)***	86 (71–108)***	83 (69–101)***	83 (68–105)***	–	–
		4 °C	101 (92–114)^‡^	98 (87–119)^‡^	100 (78–118)^‡^	97 (88–120)^‡^	92 (70–106)*	90 (76–103)***	88 (74–107)*	–	–
		− 20 °C	–	–	102 (85–117)^‡^	–	–	97 (86–114)^‡^	98 (87–109)^‡^	101 (89–110)^‡^	101 (89–109)^‡^
		− 80 °C	–	–	103 (89–118)^‡^	–	–	101 (90–117)^‡^	99 (91–112)^‡^	102 (92–112)^‡^	100 (93–116)^‡^

Afamin values are presented as median (range). In the upper part of the table, afamin plasma concentrations are given as absolute values for showing absolute recoveries; in the lower part of the table, initial afamin plasma concentrations were set at 100% to calculate percentage recoveries. Difference from respective baseline values (Wilcoxon test, not corrected for multiple comparisons): ***p < 0.001, **p = 0.001, *p < 0.05, ^‡^not significant.

**Table 2:** Characteristics of study participants in the clinical evaluation study.

Disease group	Gender (m/f)	Age (years)	Afamin (mg/L)	CRP (mg/dL)	PCT (ng/mL)	IL-6 (pg/mL)	eGFR (mL/min/1.73 m^2^)	Albumin (g/dL)	BNP (pg/mL)
Controls (n = 22)	12/10	38 (22–59)	63 (50–101)	0.1 (0.1–1.0)	0.1 (0.1–0.2)	2.0 (2.0–14)	> 90 (> 90–> 90)	4.5 (3.9–5.0)	17 (10–95)
HF (n = 15)	11/4	72 (57–85)	51 (36–89)**	0.6 (0.1–1.0)	0.1 (0.1–0.4)	4.6 (2.0–15)	> 90 (81–> 90)	3.8 (2.5–6.8)	880 (519–3109)
Pneumonia (n = 15)	13/2	57 (25–84)	26 (11–65)***	14 (2.8–29)	0.2 (0.1–2.8)	48 (7–245)	> 90 (81–> 90)	3.0 (2.2–4.0)	66 (17–96)
COPD (n = 15)	14/1	63 (51–82)	72 (8–118)^‡^	0.3 (0.1–18)	0.1 (0.1–0.4)	2.0 (2.0–16)	> 90 (85–> 90)	4.0 (3.6–4.6)	48 (10–91)
HF + Pneum. (n = 15)	12/3	80 (55–96)	25 (11–49)***	8.4 (5.8–30)	0.1 (0.1–2.2)	43 (10–1088)	87 (80–> 90)	3.1 (1.9–4.5)	864 (539–4793)
Renal disease (n = 15)	12/3	74 (55–83)	56 (29–89)^‡^	0.1 (0.1–1.0)	0.1 (0.1–0.4)	2.1 (2.0–10)	26 (9–52)	3.8 (3.2–4.6)	51 (30–96)
Sepsis (n = 15)	9/6	70 (35–83)	24 (4–55)***	25 (7.6–35)	32 (7.0–280)	1132 (112–65500)	44 (11–> 90)	2.1 (1.4–3.4)	697 (37–2575)

Data are presented as number or median (range). Difference in afamin concentrations for the control group vs. each of the disease groups (Mann–Whitney *U* test, not corrected for multiple comparisons): **p = 0.001, ***p < 0.001, ^‡^not significant.
