# Supplementary material for: Analytical characterization and clinical evaluation of an enzyme-linked immunosorbent assay for measurement of afamin in human plasma
Source: Clin Chim Acta. 2013 Oct 21;425:236–41. doi: 10.1016/j.cca.2013.08.016 (PMC3819992; doi:10.1016/j.cca.2013.08.016)
Supplement: Supplementary file 1 — Supplementary data Material and Methods.. [file mmc1.docx]

**1. Supplementary data Material and methods**

1.1. PROTEINS AND ANTIBODIES FOR THE AFAMIN ASSAY

Human afamin was purified to homogeneity from human plasma by multi-step chromatography according to a previously described procedure [1,2]. The purified protein was identified by partial sequence analysis and had the appropriate molecular mass of 65 kDa for the fully glycosylated protein. Purified afamin was used as primary protein standard for the afamin assay and for production of specific poly- and monoclonal antibodies against human afamin. The exact protein concentration of purified afamin was determined by quantitative amino acid compositional analysis on a Biochrom 20 amino acid analyzer (Cambridge, UK) equipped with a cation-exchange resin column [1]. Since previous glycan analysis of purified afamin had indicated a carbohydrate content of approximately 15%, the amino acid-derived molecular mass was multiplied by a factor of 1.15 to determine the final molecular mass of native, fully glycosylated afamin [1].

Custom-made rabbit antibodies against human afamin were obtained from Gramsch Laboratories, Schwabhausen, Germany, by immunizing New Zealand White Rabbits with human afamin, purified to homogeneity from human plasma [1,2] using complete Freund’s adjuvans (CFA). Specific anti-afamin antibodies were prepared from rabbit antisera by immunoaffinity chromatography using CNBr-activated sepharose columns to which purified human afamin was covalently bound.

Monoclonal anti-afamin antibody N13 was obtained with conventional hybridoma technology by immunizing Balb/c mice with purified human afamin, dissolved in PBS. Three mice were immunized according to the following protocol: at time 1, 100 μg/animal intraperitoneally (i.p.) with CFA; at times 1+30, 60, 90 days, 50 μg/animal i.p. at each time point, with IFA (incomplete Freund's adjuvant); at times 1+120, 121, 122 days, 50 μg/animal intravenously at each time point, without adjuvant. On day 1+123, the antibody titer was determined from a retro-orbital bleed of each animal by „quick-ELISA“ testing. This test was performed by incubating diluted mouse serum in microtiter plates, previously coated with 5 μg/ml of purified human afamin, followed by detection with peroxidase-labeled anti-mouse antibody and appropriate substrate reaction. The animal with the highest antibody titer was used on the same day for splenic fusion and creation of hybridomas. After establishing the hybridomas as stable cell cultures in 96-well culture plates with the help of mouse peritoneal macrophages as feeder cells, supernatants were screened for binding to purified human afamin again by „quick-ELISA“ testing. Of the candidate hybridomas producing antibodies with high affinity for afamin, two (N13 and M14) were selected after additional screening by immunoblotting. These two antibodies recognised only one protein band on immunoblotted purified afamin as well as human plasma, indicating high specificity for afamin. Monoclonality of antibodies was achieved by limiting dilution of hybridoma cells. Selected hybridoma cell lines were first cultivated in RPMI 1640 medium (Gibco, Life Technologies) containing 10% FCS and then transferred to serum- and protein-free culture using PFHM II medium. Under these conditions, approximately 30 mg/L immunoglobulin was found in the serum-free culture medium. Monoclonal antibodies N13 and M14 were purified from serum-free hybridoma cell culture supernatants with affinity chromatography using Protein G-coupled sepharose columns (GE Healthcare, Uppsala, Sweden). Immunoglobulins were isotyped using a commercially available kit (Monoclonal Isotyping Kit, American Qualex, San Clemente, CA, USA).

Competitive binding experiments established that each of these monoclonal antibodies recognize unique and different epitopes. Epitope mapping for the two antibodies was performed using the PepSpots peptide array technology by JPT Peptide Technologies (Berlin, Germany). 142 peptides, composed of 15 overlapping amino acids each, were bound to cellulose membranes and their recognition by antibodies N13 and M14 analyzed by dot blotting. Characterization of the epitope binding of the two monoclonal antibodies indicated that their recognition epitopes are non-linear, conformational structural epitopes. As a consequence, the ratio of available epitope to mass of protein is dependent on retention of the structure of the epitope during afamin purification; this ratio varies with each purification. To compensate for this variation, the original purified protein was maintained as a primary reference standard. Large amounts of plasma sample aliquots, obtained from fasting healthy blood donors, were used as secondary standard and calibrated to the primary reference standard. Output was defined as measurable afamin in mg per L.

1.2. BIOLOGICAL VARIATION STUDY

Twenty-two apparently healthy members of our laboratory staff (12 males and 10 females; age range, 22-59 years) were recruited for determation of the components of biological variation (intra- and inter-individual CV) for afamin plasma concentrations. Each of the volunteers had an eGFR >90 mL/min/1.73 m^2^ and plasma concentrations for CRP ≤1.0 mg/dL, for PCT ≤0.5 ng/mL, for IL6 ≤15.0 pg/mL, and for BNP ≤100 pg/mL. During the study period, all recruits maintained their usual lifestyle, and no one took any medication. Once every week for six weeks, plasma was collected from each volunteer under standardized conditions to minimize sources of pre-analytical variation. Fasting blood was obtained by conventional venipuncture between 08:00 and 10:00 in the morning by a single phlebotomist with volunteers in a sitting position. All plasma samples were aliquoted into 1.5-mL plastic tubes, frozen at –80°C and analyzed for afamin concentrations within the following two months. To keep analytical variation as low as possible, samples from any given volunteer were assayed on the same microwell plates. In addition, all analyses were carried out on the same BEP^®^ 2000 instrument by one operator. We used one set of calibrators, one lot of reagents, and single lots of any ancillary reagents and consumables. All samples were analyzed within two months of blood collection. Intra-individual biological CV (CV_I_), inter-individual biological CV (CV_G_), and the reference change value (RCV) were calculated according to the methods described by Fraser and Harris [3]. The within-run CV_A_ value was retrieved from our precision study and set to 3.3%. In brief, the total intra-individual CV (CV_TI_) was calculated from the data for each participant using the homeostatic mean of each individual. Because CV_TI_ includes analytical and biological components, the CV_I_ for each patient was obtained by subtraction using the general formula: CV_I_ = (CV_TI_^2^ – CV_A_^2^)^1/2^. The total CV (CV_T_) was then calculated by using all of the individual data sets and overall mean. The CV_I_ and CV_A_ were then subtracted from CV_T_ to determine CV_G_. Thus, we used the formula: CV_G_ = (CV_T_^2^ – CV_I_^2^ – CV_A_^2^)^1/2^. The RCV, which is the difference required for two serial measurements of afamin that have significantly changed at p <0.05, was calculated as 2.77(CV_TI_).

1.3. REFERENCE VALUES FOR AFAMIN

Reference values for the afamin assay were derived from 559 consecutive blood donors at the Red Cross organization in Linz, Austria. After peripheral venipuncture, all blood samples for afamin measurement were centrifuged and frozen in aliquots at –80°C within 4 hours. All of these samples were analyzed for afamin within two months of blood collection. Inclusion criteria were a plasma concentration for CRP ≤1.0 mg/dL, for PCT ≤0.5 ng/mL, for IL6 ≤15.0 pg/mL, and for BNP ≤100 pg/mL. Of the 559 blood donors recruited for establishing reference values, 31 did not fulfill the inclusion criteria and 528 were ultimately enrolled into the reference value study. Reference values were calculated using a non-parametric percentile method (95%, double-sided).

1.4. CLINICAL EVALUATION STUDY

For clinical evaluation of the afamin assay, we recruited 15 inpatients each with ‘heart failure (HF) without co-morbidity’, ‘pneumonia without co-morbidity’, ‘chronic obstructive pulmonary disease (COPD) without co-morbidity’, ‘HF with co-morbidity of pneumonia’, ‘renal disease without co-morbidity’, or ‘sepsis’. This was done to examine conditions suspected of influencing afamin concentrations and to disentangle possible confounders. Consequently, this is a convenient sample of highly selected patients without corresponding comorbidities. ‘HF without co-morbidity’ was defined as clinical condition with typical symptoms and signs of acute destabilized HF (e.g., dyspnea, jugular venous distension, pulmonary rales, edema of the legs or feet, etc.) according to the Framingham criteria for the clinical diagnosis of HF [4], evidence of heart enlargement by chest radiograph and/or evidence of systolic dysfunction by echocardiography, response to adequate HF therapy, and BNP plasma concentrations >500 pg/mL, but without a clinical diagnosis of pneumonia or COPD and without any laboratory evidence of inflammation (i.e., CRP ≤1.0 mg/dL, PCT ≤0.5 ng/mL, and IL6 ≤15.0 pg/mL). ‘Pneumonia without co-morbidity’ was defined as community-acquired pneumonia with symptoms and signs consistent with a lower respiratory tract infection (e.g., cough, sputum production, dyspnea, fever, auscultatory findings of abnormal breath sounds and crackles, etc.) associated with a new pulmonary opacity on chest radiograph compatible with pneumonia and response to antimicrobial therapy [5] but without a clinical diagnosis of HF or COPD and without any laboratory evidence of HF (i.e., BNP ≤100 pg/mL). ‘COPD without co-morbidity’ was defined as exacerbated disease with acute worsening of respiratory symptoms (e.g., increased dyspnea, productive cough with altered sputum, wheezing, fever, fatigue, etc.) [6], and was based on clinical history, physical examination, and spirometric criteria and response treatment with oral corticosteroids or antibiotics or both, but without a clinical diagnosis of HF or pneumonia and without any laboratory evidence of HF (i.e., BNP ≤100 pg/mL). ‘HF with co-morbidity of pneumonia’ was defined as HF with additional pneumonia according to the definitions described previously. ‘Renal disease without co-morbidity’ was defined as non-dialysis chronic kidney disease [7] associated with an eGFR <60 mL/min/1.73 m^2^ but without a clinical diagnosis of HF, pneumonia, or COPD and without any laboratory evidence of HF or inflammation (i.e., BNP ≤100 pg/mL, CRP ≤1.0 mg/dL, PCT ≤0.5 ng/mL, and IL6 ≤15.0 pg/mL). ‘Sepsis’ was defined as severe sepsis or septic shock according to the criteria of the American College of Chest Physicians/Society of Critical Care Medicine consensus conference [8]. As control group we included the healthy individuals used for the biological variation study with the results obtained from their first blood withdrawal.

**REFERENCES (supplemental data)**

[1] Jerkovic L, Voegele AF, Chwatal S, Kronenberg F, Radcliffe CM, Wormald MR, et al. Afamin is a novel human vitamin e-binding glycoprotein characterization and in vitro expression. J Proteome Res 2005;4:889-99.

[2] Voegele AF, Jerkovic L, Wellenzohn B, Eller P, Kronenberg F, Liedl KR, Dieplinger H. Characterization of the vitamin e-binding properties of human plasma afamin. Biochemistry 2002;41:14532-8

[3] Fraser CG, Harris EK. Generation and application of data on biological variation in clinical chemistry. Crit Rev Clin Lab Sci 1989;27:409-37.

[4] Senni M, Tribouilloy CM, Rodeheffer RJ, Jacobsen SJ, Evans JM, Bailey KR, Redfield MM. Congestive heart failure in the community: a study of all incident cases in Olmsted County, Minnesota, in 1991. Circulation 1998;98:2282-9.

[5] Niederman MS, Mandell LA, Anzueto A, Bass JB, Broughton WA, Campbell GD, et al. Am J Respir Crit Care Med 2001;163:1730-54.

[6] Burge S, Wedzicha JA. COPD exacerbations: definitions and classifications. Eur Respir J 2003;21:46S-53S.

[7] Levey AS, Eckardt KU, Tsukamoto Y, Levin A, Coresh J, Rossert J, et al. Definition and classification of chronic kidney disease: a position statement from Kidney Disease: Improving Global Outcomes (KDIGO). Kidney Int 2005;67:2089-100.

[8] American College of Chest Physicians/Society of Critical Care Medicine Consensus Conference: definitions for sepsis and organ failure and guidelines for the use of innovative therapies in sepsis. Crit Care Med 1992;20:864-74
